# A Rare Case of Papillary Thyroid Carcinoma Metastasizing to the Spine

**DOI:** 10.7759/cureus.85980

**Published:** 2025-06-14

**Authors:** Rami Jabareen, Zhalka Abdellatif, Ali Shalabi, Nizar Hijazi

**Affiliations:** 1 Internal Medicine, Emek Medical Center, Afula, ISR

**Keywords:** case report, lytic bone lesion, papillary thyroid cancer, spinal metastasis, thyroid neoplasms

## Abstract

Papillary thyroid carcinoma (PTC) typically presents with an excellent prognosis. Distant metastasis, particularly to the spine, is rare and associated with poorer outcomes. This report highlights the importance of considering metastatic PTC in the differential diagnosis of persistent back pain in patients with thyroid abnormalities. A 61-year-old female presented with progressive lower back pain and an enlarging neck mass. Evaluation revealed a Thyroid Imaging Reporting and Data System, Category 4 (TIRADS-4) thyroid nodule and a lytic lesion in the T10 vertebra. Biopsy confirmed papillary thyroid carcinoma with metastasis to the spine.

Spinal metastasis is an uncommon manifestation of PTC. This report underscores the need for vigilance regarding back pain in patients with a history of thyroid disease, as early detection can influence management and prevent complications. Spinal metastasis from PTC is rare but possible, even as a solitary lesion. Clinicians should consider PTC in the differential diagnosis of spinal lesions, particularly in patients with a history of thyroid abnormalities, to facilitate early diagnosis and treatment.

## Introduction

Thyroid cancer is the most common endocrine malignancy [1]. Papillary thyroid carcinoma (PTC) is the most common type of thyroid cancer (70-80% of cases) and is characterized by follicular cell differentiation and distinct nuclear features. PTC generally has the best prognosis among thyroid malignancies [2].

While PTC frequently metastasizes to regional lymph nodes, reported in 30-80% of patients, distant metastases are relatively uncommon, occurring in only 1-4% of cases. When distant spread does occur, it most commonly involves the lungs. Metastasis to the spine is extremely rare and has been sparsely documented in the literature [3].

Bone metastasis in differentiated thyroid cancers (DTC), including PTC and follicular thyroid cancer (FTC), occurs in 2-13% of cases, with a higher prevalence in FTC (7-28%) compared to PTC (1-7%) [4,5]. PTC accounts for most DTC cases but has a lower incidence of spinal metastasis, estimated at 1-7% [6]. This report presents a rare case of a metastatic spinal column lesion originating from PTC.

## Case presentation

A 61-year-old female with a history of hypertension and diabetes presented to the emergency room complaining of progressively worsening lower back pain over the past four months. She denied fever, weight loss, night sweats, neurological symptoms, or urinary incontinence.

The patient further reported that she had noticed an enlarged neck for several months before experiencing back pain. A thyroid ultrasound performed a month prior revealed a 2.60×1.7×0.11 cm Thyroid Imaging Reporting and Data System, Category 4 (TIRADS-4) mass in the right thyroid lobe and a 0.7 cm TIRADS-2 lesion in the left thyroid lobe. No lymph node enlargement was noted. In light of these findings, she underwent fine needle aspiration (FNA), with results pending at the time of her hospital admission.

**Figure 1:**
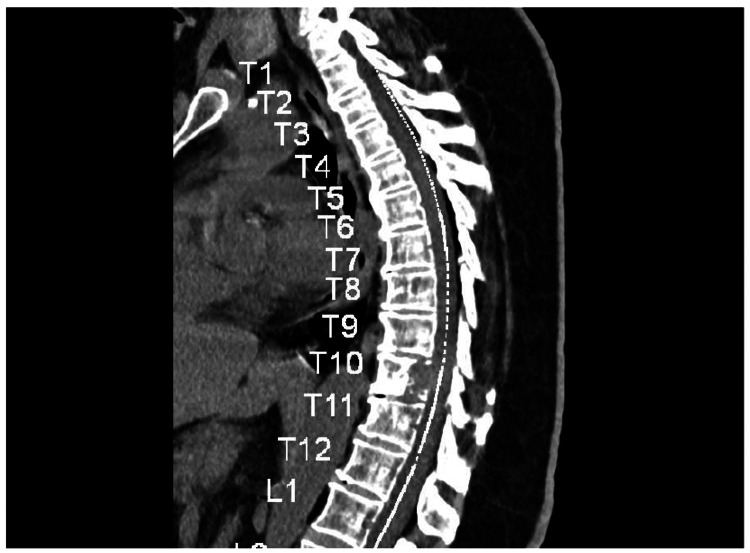
Sagittal CT spine showing a lytic lesion at T10.

Additionally, A CT scan of the spine performed a month prior revealed a lytic lesion on T10, compressing the thecal sac and causing foraminal and spinal stenosis (Figure 1). This finding raised suspicion for multiple myeloma (MM), prompting a hematologist to recommend a workup for MM.

Physical examination in the emergency department revealed that the patient appeared well, with a blood pressure of 134/101 mmHg, pulse rate of 86 beats per minute, respiratory rate of 16 breaths per minute, body temperature of 37.0°C, and oxygen saturation of 97% on room air. Further evaluation showed good power in her lower limbs and normal sensations, with no tenderness upon spinal palpation. Laboratory tests did not reveal significant abnormalities including a thyroid panel, and the workup for multiple myeloma (MM) was within normal limits (Table 1). A chest x-ray showed no lung masses or bone lesions.

**Table 1:** Laboratory investigations on admission.

Tests	Results	Reference ranges
Hemoglobin	13.5 g/dL	12.0-16.0 g/dL
White blood cell count	6.2×10⁹/L	4.0-10.0×10⁹/L
Platelet count	240×10⁹/L	150-400×10⁹/L
Serum creatinine	0.9 mg/dL	0.6-1.3 mg/dL
Serum calcium	9.4 mg/dL	8.6-10.2 mg/dL
Total protein	6.8 g/dL	6.0-8.3 g/dL
Serum albumin	4.2 g/dL	3.5-5.0 g/dL
Thyroid-stimulating hormone (TSH)	1.6 µIU/mL	0.4-4.0 µIU/mL
Free thyroxine (free T4)	1.2 ng/dL	0.8-1.8 ng/dL
Free triiodothyronine (free T3)	3.1 pg/mL	2.3-4.2 pg/mL

**Figure 2:**
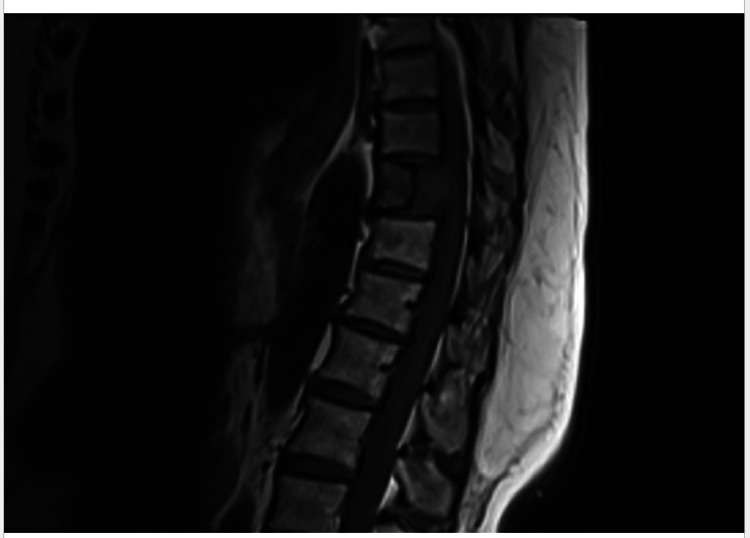
MRI showing a lytic lesion at T10.

**Figure 3:**
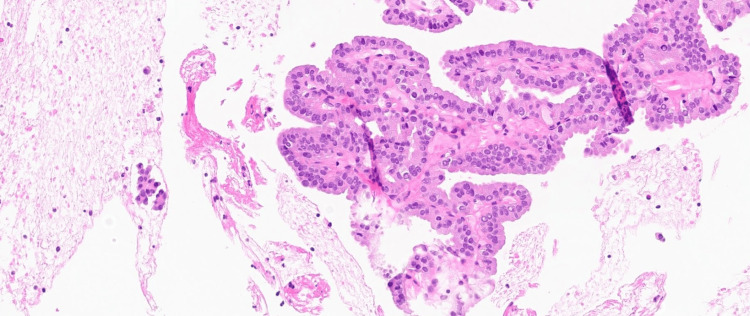
The histopathology of the spine lytic lesion.

The thyroid biopsy results were received, confirming the diagnosis of papillary thyroid carcinoma (PTC). Spine magnetic resonance imaging (MRI) revealed an expansile lytic lesion in the T10 vertebra with a mass effect but without myelopathic signal changes in the spinal cord (Figure 2). Biopsy of the vertebral mass at T10 confirmed metastasis from PTC (histopathology of the spine lesion shown in Figure 3). The patient was subsequently referred to an oncologist who planned to perform a thyroidectomy. External beam radiation therapy (EBRT) was also initiated for the spinal metastasis.

## Discussion

This report presents a rare instance of solitary spinal metastasis from PTC, a diagnosis often associated with advanced disease and multiple metastatic sites. Thyroid cancer (TC) is the most common type of endocrine malignancy, with over 90% of cases originating from differentiated thyroid cancer (DTC), which develops from follicular thyroid cells. DTC includes papillary thyroid cancer (PTC), follicular thyroid cancer (FTC), and Hürthle cell thyroid cancer, while medullary thyroid cancer originates from calcitonin-producing parafollicular C cells [7]. PTC and FTC are the most common differentiated thyroid cancers with a good prognosis, comprising 80-85% and 10-15% of all TC cases, respectively. In contrast, anaplastic thyroid cancer (ATC) is a rare, undifferentiated form (about 1% of cases) known for its aggressive behavior and poor prognosis, accounting for 15-40% of TC-related deaths [8,9].

Cervical lymph node metastasis in PTC occurs in 40-90% of cases and is a major risk factor for recurrence [10,11]. The classical variant (CVPTC) often invades the lymphatic system, while the follicular variant (FVPTC) behaves more like FTC, spreading via blood vessels to distant organs such as the lungs and bones. FVPTC is more likely to have RAS mutations and PAX8/PPARγ rearrangements [12].

The differential diagnosis for persistent back pain with lytic vertebral lesions includes primary bone tumors (osteosarcoma, chordoma), hematologic malignancies (multiple myeloma, lymphoma), infections (vertebral osteomyelitis, spinal tuberculosis), and metastatic cancers (lung, breast, prostate) [13]. Diagnostic evaluation should include a thorough physical examination, tumor markers, and imaging (MRI or PET-CT) to identify potential primary sites. Biopsy remains the gold standard for confirming malignancy [14]. In the present case, primary bone tumors, hematologic malignancies, and infections were excluded based on clinical evaluation (absence of fever, normal physical examination), laboratory results (no elevated inflammatory markers), and a negative multiple myeloma workup. A biopsy was performed to confirm the diagnosis of spinal metastasis from PTC.

Bone is the third most common site of DTC metastasis after lungs and liver [15]. Bone metastasis occurs in 7-28% of FTC cases and 12% of Hürthle cell thyroid cancer (HCTC) cases. PTC has a lower metastasis rate (1-7%) due to its preference for lymphatic spread [4].

More than 80% of bone metastases from DTC are located in the axial skeleton, which is rich in red marrow (vertebrae, ribs, and hips) [16]. Bone metastasis in spinal regions typically affects the thoracic (60-80%), lumbar (15-30%), and cervical bones (<10%) [17]. The vertebral body (85%) is the most common site. Lesions are mostly osteolytic, leading to bone destruction and spinal cord compression [17].

A bone biopsy is recommended for suspected skeletal metastasis, even in patients with known thyroid cancer, due to the possibility of another tumor type [6]. CT scans are effective for assessing bone involvement, while MRI is the gold standard for spinal metastasis imaging [17]. Spinal metastasis in PTC often indicates advanced disease and a poorer prognosis, contrasting with its typically favorable outcomes [3].

Treatment options for symptomatic or growing metastases depend on iodine uptake. If the metastases are iodine-avid, radioiodine therapy may be an option [18]. For those that do not show iodine uptake, alternative approaches include surgical resection (if possible), mutation-directed systemic therapy for unresectable disease, external beam radiation therapy (EBRT), and best supportive care [19]. Spinal metastases can lead to skeletal-related events like pathological fractures, spinal cord compression, and chronic pain. Early detection and aggressive treatment are critical [4].

This case highlights the rarity of spinal metastasis from PTC, especially as a solitary lesion. The diagnosis was confirmed with imaging and biopsy, and no neurological deficits were observed. This case emphasizes that while bone metastasis from PTC is rare, spinal metastasis should be considered in patients experiencing back pain and limb weakness. Additionally, it highlights the importance of using multiple imaging modalities to detect and assess bone metastatic lesions.

## Conclusions

Distant metastasis from papillary thyroid cancer (PTC) is observed in 1-4% of patients and infrequently affects the spine. Understanding the features and manifestations of papillary thyroid carcinoma (PTC) - particularly its potential for spinal metastasis and the role of imaging techniques - is essential for effective management and accurate prognostic assessment. This case emphasizes the need for a thorough diagnostic evaluation of persistent back pain in patients with a history of thyroid abnormalities, highlighting the potential for metastatic PTC.
